# Immediate and Gradual Withdrawal of Immunosuppression After Kidney Graft Loss Lead to Similar Outcomes

**DOI:** 10.3389/ti.2026.15642

**Published:** 2026-02-20

**Authors:** Asmaa Nabil, Nicolas Congy-Jolivet, Amandine Darres, Pierre Guy, Olivier Marion, Jean Milhes, Thomas Prudhomme, Nassim Kamar, Arnaud Del Bello

**Affiliations:** 1 Department of Nephrology and Organ Transplantation, CHU de Toulouse, Toulouse, France; 2 Centre Hospitalier et Universitaire, Université Paul Sabatier Toulouse III, Toulouse, France; 3 Laboratory of Immunology, Biology Department, Centre Hospitalier et Universitaire (CHU) de Toulouse, Toulouse, France; 4 Institute of Metabolic and Cardiovascular Diseases (I2MC), Institut National de la Santé et de la Recherche Médicale (INSERM) U1297, University of Toulouse 3, Toulouse, France; 5 Department of Urology and Renal Transplantation, CHU de Toulouse, Toulouse, France; 6 Toulouse Institute for Infectious and Inflammatory Diseases (Infinity), INSERM UMR1043-CNRS 5282, Toulouse, France

**Keywords:** allograft nephrectomy, allo-sensitization, graft failure, immunosuppression, intolerance syndrome

## Abstract

The management of immunosuppression in dialysis patients with a failed kidney transplant remains a pending question, and different approaches to immunosuppression weaning have been proposed. We conducted a retrospective study of patients who experienced a graft failure, and compared the rates of immune and non-immune events, according to different modalities of immunosuppression withdrawal. Two hundred and eighteen patients were included. During the follow-up (45 (20–80) months post-graft failure), 53 patients (24.3%) experienced an intolerance syndrome. The time between graft failure and the occurrence of intolerance syndrome was 6 (3–13) months. Immunosuppression withdrawal was associated with the occurrence of intolerance syndrome. However, regarding the immunosuppression withdrawal modality, only a steroid cessation during the first 3 months post graft failure was independently associated with an earlier occurrence of intolerance syndrome [HR = 1.91, 95%CI (1.08–3.38), *p* = 0.025], while a longer time between transplantation to graft failure was independently associated with a delayed occurrence of intolerance syndrome [HR = 0.99, 95%CI (0.98–0.99), *p* = 0.009]. The immunosuppression withdrawal modality after graft failure didn’t have an impact on infections and cardiovascular complications. Although discontinuation of immunosuppression strongly influences the occurrence of intolerance syndrome, immunosuppression withdrawal modality itself does not appear to.

## Graphical Abstract

**Figure:**
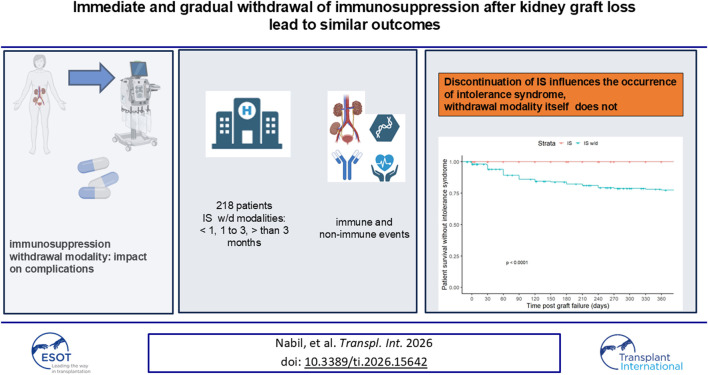

## Introduction

Anti-HLA sensitization remains a significant barrier in kidney transplantation because of the risk of antibody-mediated rejection in the setting of preformed DSA [1]. Despite the attention paid to pregnancy or blood transfusion, the rate of hypersensitized recipients on the waiting list didn’t decrease over time [2–4], and a majority of these patients are candidates for a retransplantation [3, 4]. Although anti-HLA antibodies could develop after transplantation, the incidence of *de novo* DSA detection is less than 10% in recipients with a functioning graft [5, 6]. However, an intense allo-sensitization is observed after patients return to dialysis and stop immunosuppression while waiting for another transplant. Augustine and colleagues found an increase of highly sensitized recipients (defined as a cPRA≥80%) from 21% to 68% between the time of transplant failure and 2 years later [7]. Billen and colleagues found in a cohort of 56 patients that 16% presented detectable *de novo* DSA at graft failure, but the proportion increased to more than 80% after ceasing immunosuppression [8]. A majority of anti-HLA antibodies detected after graft loss are considered to be donor-specific at the epitope level [9].

After patients return to dialysis and immunosuppression is reduced or stopped, an allograft nephrectomy could be required in case of graft rejection occurring in a failed transplant (the so-called “intolerance syndrome”), graft malignancy, persistent C-reactive protein or to create space for another transplant [10]. The incidence of allograft nephrectomy in this setting can be as high as 30% [11], and appears to be more frequent during the first 6 months post-dialysis initiation [11–15]. Allograft nephrectomy is associated with non-immunological complications (mainly infections) in up to 30% [16] of case, including death. Furthermore, it is also responsible for an increase in anti-HLA antibodies occurrence, mainly in less sensitized patients [9, 17].

The management of immunosuppression in dialysis recipients with a failed transplant remains a pending question. Some but not all reports have suggested that maintaining immunosuppression could increase hospitalizations, and major adverse events such as infections, cardiovascular events, or cancers [12, 18–25]. Hence, currently, except for patients with a planned retransplantation from a living donor, most transplant societies propose ceasing immunosuppression during the first year after graft failure [10, 26, 27]. However, an immediate or progressive stop of immunosuppression over 1 year after graft failure was not assessed until now.

The present study first aimed to compare the incidence of intolerance syndrome after return to dialysis, according to the modality of immunosuppression withdrawal. The secondary aims were to compare the incidence of allo-sensitization, infection, neoplastic, and cardiovascular complications following graft failure according to the type of immunosuppressive withdrawal strategy.

## Patients and Methods

### Patients

This retrospective study obtained approval from the Toulouse IRB (RC31/21/01/54).

The study was conducted using our Institution Electronic Medical Records. All adult kidney transplant recipients who experienced graft failure between 01.01.2008 and 31.12.2022 were screened for inclusion (n = 418). The date of graft failure was defined as the date of starting hemodialysis or peritoneal dialysis.

Patients were excluded in case of preemptive transplantation (n = 3), combined transplantation that required the maintenance of immunosuppression (n = 23), need for graft nephrectomy of a functioning transplant (surgical complication, n = 31), immediate graft nephrectomy (and hence immunosuppression withdrawal) after transplantation (vascular complications during the first 8 days post transplantation, n = 125), and loss of follow-up immediately (<1 month after return to dialysis, n = 18). The last follow-up was considered as last medical appointment until July 01 2023. Finally, 218 patients were included in the study.

### Immunosuppression

The time for immunosuppression discontinuation was defined as the time between the date of graft failure and the date of the last prescription recorded of any treatment, including calcineurin inihibitors (CNI), antimetabolites, mammalian target of rapamycin (mTOR) inhibitors, belatacept, and steroids. Since several recommendations were proposed, immunosuppressant discontinuation modalities were at the clinician’s discretion:

- Modality 1: brutal CNI and/or antimetabolites/mTOR inhibitors discontinuation at return to dialysis (no immunosuppression except steroids after 1 month post-graft failure).
- Modality 2: CNI and antimetabolites/mTOR inhibitors were maintained at the same dose until they were discontinued between 1 and 3 months post-graft failure.
- Modality 3: CNI and antimetabolites/mTOR inhibitors maintained at the same dose until they were discontinued more than 3 months post graft failure.

All patients had been given a low dose of steroids until graft failure (5 mg/day). After graft failure, steroids were converted to hydrocortisone or stopped (with or without ACTH stimulation test), at the clinician’s discretion (in the absence of recommendations).

### Outcomes

The primary outcome of this study was the occurrence of intolerance syndrome after return to dialysis. Intolerance syndrome was defined as the occurrence of graft pain with or without gross hematuria, fever, refractory anemia, or elevated C reactive protein (after exclusion of infection or cancer) [14]. Cases were identified and reviewed by 2 senior nephrologists from electronic medical records (AN, ADB). Secondary outcomes included the following: infection episodes requiring hospitalization, and cardiovascular complications (number, type, and time from return to dialysis to event were obtained from electronic medical records), allo-sensitization (as defined by the calculated panel reactive antibody cPRA with the vPRA online tool^1^. Opportunistic infections were defined as previously published [28, 29]. Major Adverse Cardiovascular Events (MACE) were defined as previously published [30]. The presence of anti-HLA antibodies was assessed every 6 months after return to dialysis in patients eligible for retransplantation, and detected using the Lifecodes™ single antigen technology (LMX deluxe Immucor, Gateway Drive, GA). The Lifecodes™ single antigen (LSA class I/II) determined the specificity of class I HLAs in A/B/Cw and class II in DR/DQ/DP IgG antibodies in the recipients’ sera according to the manufacturer’s instructions. The presence and specificity of antibodies were then detected, and the mean fluorescence intensity for each sample in each bead was evaluated. A mean fluorescence intensity value of >1,000 was considered positive.

### Statistical Analyses

Reported values represent the means (±SD) or medians (IQR). Quantitative variables were compared using the student T-test or Mann-Whitney non-parametric test if appropriate. Categorical variables are expressed as percentages and compared between groups using the chi-squared tests or, if appropriate, Fisher’s exact test. A *p*-value of <0.05 was considered statistically significant.

Analyses were performed with R, version 4.2.2 (R Development Core Team, Vienna, Austria).

Missing data represented less than 10% in each variable of the dataset (medical history [donor age, initial nephropathy, diabetes at dialysis initiation], initial immunosuppression [CNI type]) and were imputed (excluding outcomes) using the MICE package.

We used Kaplan-Meier curves (with log-rank tests) and univariate and multivariate Cox models (including all statistically significant variable in univariate analysis, and variables known to be clinically relevant such as donor and recipient age or class-I and II HLA mismatches) with backward elimination to estimate the association between the different immunosuppressant withdrawal modalities and the outcomes (intolerance syndrome, cardiovascular diseases, infections). The proportional hazards assumption was verified using Schenfeld residuals.

Survival without intolerance syndrome was analyzed using Cox proportional hazards models with time-dependant covariates. Time was measured from dialysis initiation until the occurrence of intolerance syndrome, or last-follow-up. The use of immunosuppression was modeled as a time-dependant covariate: patients contributed to the “immunosuppression” category until treatment discontinuation (except steroids), and to the “immunosuppression withdrawal” thereafter. This approach allowed patients to contribute risk time to both exposure groups according to their actual status during follow-up.

Survival analyses were performed with the survminer and survival packages.

## Results

### Description of the Cohort

Baseline characteristics of the included patients are described in Table 1. The majority of patients were male, first kidney transplant recipients, non-HLA sensitized at transplantation, and did not receive a T-cell depleting agent at the time of transplantation. The median follow-up period between transplantation and graft failure was 45 (20-80) months. The primary causes of graft loss were chronic antibody-mediated rejection and interstitial fibrosis/tubular atrophy (IF/TA). Most patients began dialysis with a triple therapy regimen that included calcineurin inhibitors, mycophenolate, and steroids. The median follow-up period between graft failure and the last follow-up was 22.7 (10.9; 38.6) months. The median follow-up period between the cessation of immunosuppression and the last follow-up was 21.4 (8.8; 45.8) months. Except steroids, immunosuppression withdrawal was achieved in less than 1 month for 84 patients [median 0.0 (0.0; 0.0) months], between 1 and 3 months for 80 patients [median (1.1 (1.0; 2.9) months], and in more than 3 months for 54 patients [median 5.9 (4.9; 10.8) months]. With respect to steroids, they were stopped in 11, 47 and 160 patients, within the first month, between 1 and 3 months, and after 3 months after graft failure, respectively.

**TABLE 1:** Baseline characteristics of the cohort.

Variables	Modality- 1 <1 month	Modality-2 [1-3 months]	Modality – 3 >3 months	*P*-value
Number of patients	**84**	**80**	**54**	​
Gender, male (%)	52 (61.9)	49 (61.3)	31 (57.4)	0.86
Recipient age at transplantation, mean (SD)	53.2 (15.1)	52.5 (13.9)	49.3 (17.2)	0.32
Initial nephropathy (%) Interstitial - genetic Glomerular Diabetic Vascular Other-uknown	22 (26.2) 28 (33.3) 8 (9.5) 14 (16.7) 12 (14.3)	27 (33.8) 26 (32.5) 10 (12.5) 11 (13.8) 6 (7.5)	20 (37) 14 (25.9) 5 (9.3) 11 (20.4) 4 (7.4)	0.67
Number of previous transplantations (%) 0 1 2 3 or more	63 (75.0) 17 (20.2) 2 (2.4) 2 (2.4)	59 (73.8) 15 (18.9) 5 (6.3) 1 (1.3)	47 (87.0) 7 (13.0) 0 0	0.40
Donor age at transplantation, mean (SD)	57.6 ± 14.8	57.5 ± 17.6	53.7 ± 19.1	0.22
T cell depleting agent at induction (%)	24 (28.6)	31 (38.8)	13 (24.1)	0.16
HLA A/B/DR/DQ mismatches, mean (SD)	4.88 (1.63)	5.01 (1.62)	4.83 (1.9)	0.81
cPRA at transplantation, median (IQR) Class I Class II	0 (0; 22) 0 (0; 50)	0 (0; 31.5) 0 (0; 60)	0 (0; 30) 0 (0; 32)	0.96 0.44
Time between transplantation – return to dialysis, in months, mean (SD)	46.8 (35.9)	51.8 (38.2)	60.9 (38.6)	0.10
Diabetes at return to dialysis (%)	12 (14.3)	11 (13.8)	6 (11.1)	0.86
Cause of graft loss (%) Chronic antibody mediated rejection Chronic T cell mediated rejection Reoccurrence of initial disease PVAN Chronic infection IFTA	21 (25) 16 (19.0) 7 (8.3) 9 (10.7) 3 (3.6) 28 (33.4)	30 (37.5) 14 (17.5) 2 (2.5) 5 (6.2) 1 (1.3) 28 (35.0)	20 (37.0) 3 (5.6) 3 (5.6) 5 (9.3) 1 (1.9) 22 (40.6)	0.26
Immunosuppression at return to dialysis (%) CNI MPA mTOR inhibitors Belatacept Steroids	69 (82.1) 52 (61.9) 20 (23.8) 13 (15.5) 84 (100)	78 (97.5) 49 (61.3) 12 (15) 3 (3.8) 80 (100)	51 (94.4) 27 (50) 13 (24.1) 10 (18.5) 54 (100)	0.002 0.32 0.29 0.016 >0.99
Steroids withdrawal (%) <1 month 1-3 months >3 months	8 (9.5) 22 (26.2) 54 (64.3)	0 22 (27.5) 58 (72.5)	3 (5.6) 3 (5.6) 48 (88.9)	0.001
Time between dialysis initiation – last follow-up, months, median (IQR)	22.5 (11.1; 51.7)	20.6 (9; 43.4)	30.5 (13.8; 42.4)	0.83
Time between IS withdrawal – last follow-up, months, median (IQR)	22.5 (11.1; 51.7)	20.0 (7.9; 42.8)	21.8 (2.8; 37.9)	0.05

Abbreviations: SD, standard deviation; cPRA, calculated panel reactive antibodies; IQR, interquartile range; PVAN, polyoma virus associated nephropathy; IFTA, interstitial fibrosis tubular atrophy; CNI, calcineurin inhibitors; MPA, mycophenolic acid; mTOR, mammalian target of rapamycin.

### Intolerance Graft Syndrome and Allo-Sensitization

**FIGURE 1:**
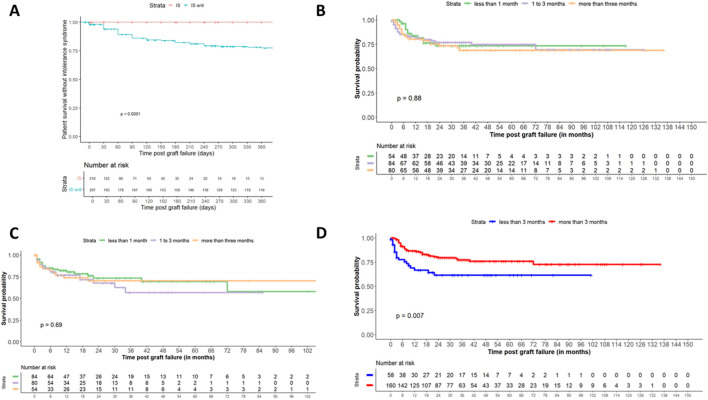
Occurrence of intolerance syndrome according to immunosuppression withdrawal modality **(A)**. Patient survival free from intolerance syndrome according to immunosuppression exposition. Patients contributed to the “immunosuppression” category until treatment discontinuation (except steroids), and to the “immunosuppression withdrawal” thereafter. The *p*-value in the Kaplan-Meier curve was tested the log-rank test. **(B)**. Patient survival free from intolerance syndrome after graft failure. The *p*-value in the Kaplan-Meier curve was tested the log-rank test. **(C)** Patient survival free from intolerance syndrome after immunosuppression withdrawal (apart from steroids). The *p*-value was tested the log-rank test. **(D)** Patient survival free from intolerance syndrome after steroid withdrawal (less or more than 3 months). The p-value was tested the log-rank test.

During the follow-up, fifty-three patients (24.3%) experienced an intolerance syndrome (Table 2). The time between graft failure and the occurrence of intolerance syndrome was 6 (3; 13) months. The time of intolerance syndrome and immunosuppression discontinuation was 1 (2; 9) months. All patients received a course of steroids for 1 week at 1 mg/kg. The treatment was effective and sufficient in 3 cases, without reoccurrence after a decrease in steroids over 1 month. The remaining 50 patients required a surgical graft nephrectomy (n = 43) or a renal artery embolization (n = 7). The impact of immunosuppression cessation on the development of intolerance syndrome was modelled using a Cox proportional hazards model with immunosuppression therapy as a time-dependant covariate. We observed that IS cessation was associated with the occurrence of intolerance syndrome [HR: 11.41, 95%CI (4.20–31.03), *p* < 0.0001, Figure 1A]. This remained true after adjustment for donor and recipient age, and time between transplantation and graft failure [aHR: 10.37, 95%CI (3.79–28.42), p < 0.0001]. When considering the modality of cessation of all immunosuppressants except steroids, we did not observe a difference for the rate or the time to intolerance syndrome after graft failure (Figures 1B,C) or after IS cessation (Figures 1C,D; Table 1). However, a steroid cessation during the first 3 months post graft failure was independently associated with a reduced time between graft failure and the occurence of intolerance syndrome [aHR = 2.31, 95% CI (2.13–2.50), *p* < 0.001], while a longer time between transplantation to graft failure was independently associated with a prolonged time between graft failure and the occurrence of intolerance syndrome [aHR = 0.99, 95% CI (0.98–0.99), *p* < 0.001], after adjustment for donor and recipient age, number of HLA mismatches, modality of cessation of immunosuppressants except steroids (Figures 1C,D; Table 1; Supplementary Tables 1, 2).

**TABLE 2:** Baseline characteristics of the cohort who presented or not an intolerance syndrome.

Variables	Intolerance syndrome	No intolerance syndrome	*P*-value
Number of patients	**53**	**165**	​
Gender, male (%)	29 (54.7)	103 (62.4)	0.18
Recipient age at transplantation, mean (SD)	50.0 ± 15.0	52.3 ± 15.1	0.18
Initial nephropathy (%) Interstitial - genetic Glomerular Diabetic Vascular Other-uknown	18 (34.0) 16 (30.2) 6 (11.3) 11 (20.7) 2 (3.8)	51 (30.9) 52 (31.5) 17 (10.4) 25 (15.1) 20 (12.1)	0.45
Number of previous transplantations (%) 0 1 2 3 or more	39 (73.6) 11 (20.7) 2 (3.8) 1 (1.9)	130 (78.8) 28 (17.0) 5 (3.0) 2 (1.2)	0.37
Donor age at transplantation, mean (SD)	53.8 ± 18.0	57.4 ± 16.6	0.21
T cell depleting agent at induction (%)	18 (33.9)	50 (30.3)	0.74
HLA A/B/DR/DQ mismatches, mean (SD)	4.9 ± 1.6	4.9 ± 1.7	0.35
cPRA at transplantation, median (IQR) Class I Class II	0 (0; 22) 0 (0; 8)	0 (0; 30) 0 (0; 50)	0.41 0.10
Time between transplantation – return to dialysis, in months, mean (SD)	38.6 ± 35.4	56.5 ± 37.5	0.38
Diabetes at return to dialysis (%)	8 (15.1)	21 (12.7)	0.83
Cause of graft loss (%) Immune related, yes Chronic antibody mediated rejection Chronic T cell mediated rejection Other, yes Reoccurrence of initial disease PVAN Chronic infection IFTA	26 (49.0) 18 (34.0) 8 (15.0) 27 (51) 0 0 2 (3.8) 25 (47.2)	78 (47.3) 53 (32.1) 25 (15.1) 87 (52.7) 12 (7.3) 19 (11.6) 3 (1.8) 53 (32.1)	0.95
Immunosuppression at return to dialysis (%) CNI MPA mTOR inhibitors Belatacept Steroids	49 (92.4) 32 (60.4) 9 (17.0) 6 (11.3) 53 (100)	149 (90.3) 96 (58.2) 36 (21.8) 20 (12.1) 165 (100)	0.97
Steroids withdrawal (%) <1 month 1-3 months >3 months	2 (3.8) 17 (32.0) 34 (64.2)	9 (5.4) 30 (18.2) 126 (76.4)	0.10

Abbreviations: SD, standard deviation; cPRA, calculated panel reactive antibodies; IQR, interquartile range; PVAN, polyoma virus associated nephropathy; IFTA, interstitial fibrosis tubular atrophy; CNI, calcineurin inhibitors; MPA, mycophenolic acid; mTOR, mammalian target of rapamycin.

**FIGURE 2:**
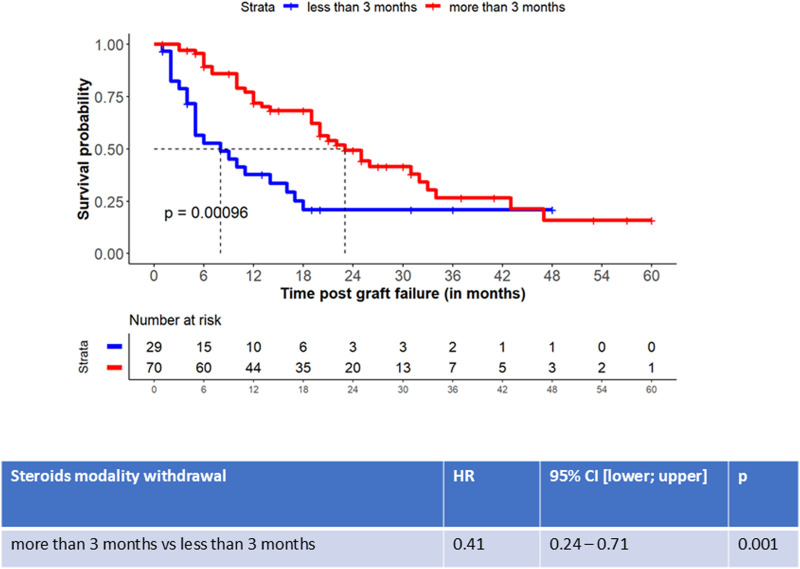
Occurrence of *de novo*-Donor Specific Antibodies according to the timing of steroid withdrawal. Patient survival free from *de novo* DSA after graft failure. The p-value was tested the log-rank test. Association between the steroid withdrawal modality after graft failure and *de novo* DSA occurrence. The association was tested by using a univariate Cox proportional hazards model.

**FIGURE 3:**
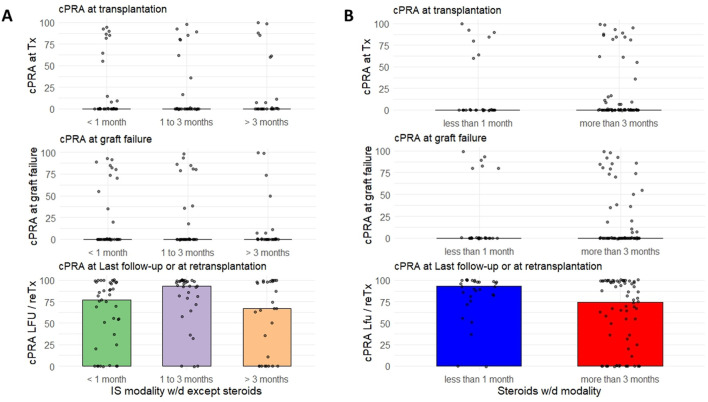
Evolution of the cPRA at transplantation, graft failure and last-follow-up, according to **(A)** immunosuppression withdrawal modality except steroids, and **(B)** steroid withdrawal modality. Abbreviation: cPRA, calculated Panel Reactive Antibodies; Tx, Transplantation; LFU, Last Follow-up; reTx, re-Transplantation; w/d, withdrawal.

After graft failure, 99 patients (45.4%) were considered for a retransplantation and screened for anti-HLA sensitization. 56 of the 99 (58.6%) had developed *de novo* DSA at last follow-up [21 (9; 39) months post-graft failure]. We assessed the role of immunosuppression cessation modality on the occurrence of post-graft failure allo-sensitization and found that a delayed withdrawal of immunosuppression other than steroids did not affect the occurrence of anti-HLA DSA (Figure 2; Supplementary Table 2). However, although a delayed steroids withdrawal was associated with a reduced occurrence of DSA (HR 0.41, 95% CI [0.24–0.71] *p* = 0.001, Figure 2; Supplementary Table 2), we did not observe any difference on cPRA values at last follow-up, according to immunosuppression discontinuation modality (Figure 3). 12 of the 99 patients received an allograft nephrectomy: 7 patients presented a DSA before the nephrectomy, 2 patients presented *de novo* DSA after the nephrectomy, and 3 patients did not presented DSA pre- or post nephrectomy. The cPRA increased before the nephrectomy (median cPRA: 0 (0; 93) at transplantation, 0 (0; 93) at graft failure, 52 (0; 100) before the nephrectomy [3.6 (0.9; 32.6) months post graft failure and 1.0 (0.0; 8.7) months before the nephrectomy], and after the nephrectomy (median cPRA: 96 (36; 100) at last follow-up, after 50.4 (5.7; 119.9) months post graft failure. Forty-nine (22.5%) patients received another transplantation during the follow-up. We did not observe any difference regarding the different modalities of immunosuppression withdrawal and the occurrence of retransplantation (retransplantation according to immunosuppression withdrawal modalities except steroids, withdrawal <1 month as the reference group, HR = 1.32, 95%CI [0.93; 1.87], *p* = 0.19; retransplantation according to steroid withdrawal modality [withdrawal <3 months as the reference group, HR = 0.82, 95% CI (0.47; 1.43), *p* = 0.99]).

### Infection and Cardiovascular Complications

**FIGURE 4:**
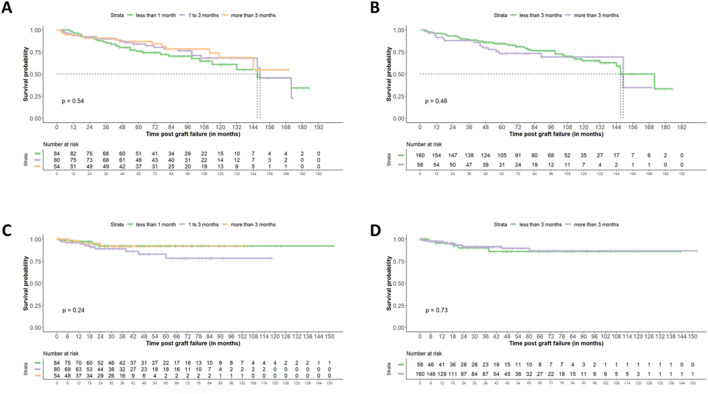
Occurrence of infections and Major Adverse Cardiovascular Events (MACE) according to immunosuppression withdrawal **(A)**. Patient survival free from infection requiring a hospitalization after graft failure. The p-value was tested the log-rank test. **(B)** Patient survival free from infection requiring a hospitalization after graft failure according to the steroid withdrawal modality. **(C)** Patient survival free from MACE requiring an hospitalization after graft failure. The p-value was tested the log-rank test. **(D)** Patient survival free from MACE requiring an hospitalization after graft failure according to the steroid withdrawal modality.

Sixty-three out of the 218 patients (28.9%) experienced at least one episode of infection requiring hospitalization during the follow-up, (19 of the 63 (30.2%) occurred in patients that were still under immunosuppression). Among them, 20 patients (31.7%) required an intensive care unit hospitalization at least once. Twelve patients (including 5 who were still under immunosuppression) developed at least one opportunistic infection [cytomegalovirus syndrome (n = 6), HSV-2 viremia (n = 1), invasive pulmonary aspergillosis (n = 1) and invasive non-aspergillosis mold (n = 1), candidemia (n = 2), cryptosporidiosis induced diarrhea (n = 1)]. We did not find any difference regarding the type and length of immunosuppression after graft failure and the occurrence of infectious complications (Figures 4A,B; Supplementary Table 4).

Eighteen patients (8.3%) experienced at least one MACE during the follow-up (Supplementary Table 5). We did not find any difference regarding the occurrence of MACE and type and length of immunosuppression during dialysis (Figures 4C,D).

Fifty-nine patients (27.0%) died during the follow-up (Supplementary Figure 1A,B; Supplementary Table 6). We did not observe any difference regarding different modalities of immunosuppression withdrawal and death (death according to immunosuppression withdrawal modalities (except steroids): <1 month: 30/84, 1–3 months 16/80, more than 3 months 13/54, *p* = 0.07; death according to steroid withdrawal modality: <3 months, 15/58, >3 months 44/160, *p* = 0.81).

## Discussion

Although patients with a failing graft represent an increasing proportion among the dialysis population [27], return to dialysis remains a critical period. At that time, the management of immunosuppression is quite complex. Indeed, maintaining immunosuppression is considered to be risky because of the limited immediate expected benefit compared with potential complications. A higher risk for cardiovascular and infection disorders have been reported, leading to an increased risk of death compared to patients with poor allograft function, or incident dialysis patients without a history of transplantation [31–35]. However, clinicians may be prompted to pursue immunosuppressants in order to reduce the risk of allo-sensitization. Our study is in line with previous studies and shows that the risk of allo-sensitization is not very high during the graft functioning period [5, 6] and as long as the patient remains on immunosuppression [19], while returning to dialysis after immunosuppression withdrawal remains a high risk period when allo-sensitization occurs [7–9, 11, 17, 36–38], that represents a significant limitation for future transplantation. Moreover, intolerance syndrome, that is related to immunosuppressants’ withdrawal, is a major complication that could lead to the need for allograft nephrectomy or arterial embolization that may induce other complications. Available guidelines to manage immunosuppression in those patients remain elusive [10, 26].

In the present retrospective study, we assessed the impact of different strategies of immunosuppression withdrawal on the occurrence of intolerance syndrome. The incidence of intolerance syndrome in our cohort was 24.3%, and occurred early after graft failure (median 6 (3; 13) months post-graft failure), which is concordant with previous studies [7, 11, 39]. We noted a strong association between immunosuppression withdrawal and intolerance syndrome occurrence. However, we did not observe any difference in the incidence or the timing of intolerance syndrome or anti-HLA sensitization after graft failure and the modality of immunosuppression withdrawal, except for steroid management which was associated with a delay in the occurrence of this complication. This is in line with previous retrospective [23, 40, 41] and prospective studies [19, 42]. How to handle steroids after kidney graft failure is often not described and not considered in the strategy of management of immunosuppression in this critical period [10, 27]. However, our study suggest that the impact of low-dose steroids in this setting could be non-zero. Indeed, we found that patients who stopped their steroids during the first 3 months could present an intolerance syndrome earlier, and independently of the way of management of other immunosuppressants, i.e., CNIs, mTOR inhibitors and MPA. In a retrospective study of 89 patients who returned to dialysis, Garg and colleagues found that steroid continuation was associated with significantly lower odds of developing an absolute increase of allo-sensitization [43]. Low-dose steroids alter T-cell and antibody mediated responses [44–46]. Discontinuation of low-dose steroids was previously associated with immune reconstitution inflammatory syndrome after a prolonged course of treatment [47]. Taken together, these observations suggest that (i) reduced doses, without monitoring drug dose levels, for a limited period (i.e., not prolonged until retransplantation) is inefficient to prevent allo-sensitization or intolerance syndrome after graft failure, and (ii) in cases where early immunosuppression discontinuation is proposed, a brutal withdrawal of steroids could participate in the development of intolerance syndrome and allo-sensitization complications. Nonetheless, these findings should be interpreted as exploratory. Unmeasured factors not included in our analysis may have contributed to early steroid discontinuation and could therefore have introduced residual confounding. Validation of these results in future external cohorts will be important to confirm their generalizability. We didn’t find an impact of a gradual maintenance immunosuppression withdrawal on the rate of infections requiring hospitalization, opportunistic infections, and cardiovascular complications in dialysis patients during follow-up. This could be explained by the low doses of CNIs/antimetabolites and early drug discontinuation. Some [23, 40], but not all [12], retrospective studies did not observe a higher risk for infections or MACE in the same seting. However, other adverse effects (e.g., hypertension, dyslipidemia, diabetes, etc…) that alter the long-term cardiovascular health should be kept in mind.

Our study presents several limitations. This is a retrospective, monocentric study. Immunosuppressants’ doses and trough levels were not assessed. The number of intolerance syndromes included was small, limiting our ability to included multiple parameters in our multivariate analyses. Nonetheless, this helped us to obtain a granular assessment of immunosuppression management in these patients, particularly regarding the management of steroids. These results could guide the development of future large prospective clinical study.

In conclusion, although discontinuation of immunosuppression strongly influences the occurrence of intolerance syndrome, the modality of immunosuppression withdrawal according to available recommendations in patients with a failed graft do not prevent the occurrence of intolerance syndrome, or allo-sensitization. Only a brutal, early (<3 months) withdrawal of steroids seems to reduce the time between graft failure and the development of intolerance syndrome. Our data suggest further evaluation of the impact of current expert’s opinion-based guidelines.

## Data Availability

The data analyzed in this study is subject to the following licenses/restrictions: Available upon request. Requests to access these datasets should be directed to AD, delbello.a@chu-toulouse.fr.

## Footnotes

1
www.etrl.org/vPRA
