# Supplementary material for: Immediate and Gradual Withdrawal of Immunosuppression After Kidney Graft Loss Lead to Similar Outcomes
Source: Transpl Int. 2026 Feb 20;39:15642. doi: 10.3389/ti.2026.15642 (PMC12963015; doi:10.3389/ti.2026.15642)
Supplement: Supplementary file 1 [file Supplementaryfile1.docx]

**Supplementary Information**

**Table 1. Univariate and multivariate analyses of factors associated with the occurrence of intolerance syndrome during the follow-up after graft failure.**

| Variable | HR | 95% CI (lower- upper) | p-value | HR | 95% CI (lower- upper) | p-value |
| --- | --- | --- | --- | --- | --- | --- |
| Recipient age at transplantation | 0.99 | 0.97 – 1.01 | 0.28 |  |  |  |
| Donor age at transplantation | 0.99 | 0.97 – 1.00 | 0.20 |  |  |  |
| HLA A,B,DR,DQ mismatches | 0.98 | 0.83 – 1.15 | 0.80 |  |  |  |
| Previous transplantations | 1.35 | 0.74 – 0.89 | 0.20 |  |  |  |
| Induction therapy with at transplantation with a depleting agent | 1.07 | 0.60 – 1.89 | 0.82 |  |  |  |
| Graft failure from immunological cause | 1.00 | 0.59 – 1.73 | 0.98 |  |  |  |
| Time between transplantation – graft failure | 0.99 | 0.98 – 0.99 | 0.003 | 0.99 | 0.98 – 0.99 | 0.001 |
| Time between IS withdrawal – end of follow-up | 1.02 | 1.01 – 1.03 | <0.0001 |  |  |  |
| Infection after graft failure* | 0.92 | 0.47 – 1.78 | 0.80 |  |  |  |
| IS withdrawal <1month vs 1-3 months | 1.13 | 0.60 – 2.10 | 0.70 |  |  |  |
| IS withdrawal 1-3 months vs > 3 months | 1.20 | 0.84 – 0.58 | 0.63 |  |  |  |
| IS withdrawal < 1 month vs > 3 months | 0.95 | 0.50 – 1.10 | 0.89 |  |  |  |
| CNI withdrawal < 1 month vs 1-3 months | 1.16 | 0.61 – 2.19 | 0.65 |  |  |  |
| CNI withdrawal < 1 month vs > 3 months | 1.00 | 0.47 – 2.11 | 0.99 |  |  |  |
| CNI withdrawal 1-3 month vs > 3 months | 0.86 | 0.41 – 1.82 | 0.70 |  |  |  |
| MMF/MPA withdrawal < 1 month vs 1-3 months | 1.53 | 0.72 – 3.26 | 0.26 |  |  |  |
| MMF/MPA withdrawal < 1 month vs > 3 months | 1.54 | 0.45 – 5.20 | 0.50 |  |  |  |
| MMF/MPA withdrawal 1-3 months vs > 3 months | 0.99 | 0.28 – 3.57 | 0.99 |  |  |  |
| Steroid withdrawal <3 months vs > 3 months | 2.1 | 1.21 – 3.74 | 0.0008 | 2.31 | 2.13 – 2.50 | <0.001 |

Abbreviation: IS, immunosuppression

*Only infection requiring an hospitalization were included. Only infection that occurred before the intolerance syndrome were included.

In multivariate analysis, results were adjusted for donor and recipient age, HLA-A,B,DR,DQ mismatches, modality of cessation of immunosuppressants except steroids.

Recipient and Donor age, Time between immunosuppression withdrawal -end of follow-up or Time between transplantation – graft failure were tested as continuous variables.

**Supplementary Table 2 (A-C):**

1. Univariate Cox regression model assessing the association between the immunosuppression withdrawal modality (apart from steroids) after graft failure and intolerance syndrome occurrence.

| IS modality withdrawal | HR | 95% CI [lower; upper] | p |
| --- | --- | --- | --- |
| During the first month vs 1 to 3 months | 1.13 | 0.60 – 2.10 | 0.70 |
| During the first month vs more than 3 months | 0.95 | 0.50 - 1.10 | 0.89 |
| 1 to 3 months vs more than 3 months | 1.20 | 0.84 – 0.58 | 0.63 |

1. Univariate Cox regression model assessing the association between the immunosuppression withdrawal modality (apart from steroids) after graft failure and intolerance syndrome occurrence.

| IS modality withdrawal | HR | 95% CI [lower; upper] | p |
| --- | --- | --- | --- |
| During the first month vs 1 to 3 months | 1. 31 | 0.71 – 2.42 | 0.70 |
| During the first month vs more than 3 months | 1.13 | 0.55 – 2.31 | 0.89 |
| 1 to 3 months vs more than 3 months | 1.16 | 0.57 – 2.37 | 0.63 |

1. Univariate Cox regression model assessing the association between steroid withdrawal modality after graft failure and intolerance syndrome occurrence.

| Modality of steroid withdrawal | HR | 95% CI [lower; upper] | p |
| --- | --- | --- | --- |
| During the first 3 months vs more than 3 months | 2. 1 | 1.21 – 3.74 | 0.008 |

**Supplementary Table 3**

**Univariate analysis of factors associated with the occurrence of Donor Specific Antibodies during the follow-up after graft failure.**

| Variable | HR | 95% CI (lower- upper) | p-value |
| --- | --- | --- | --- |
| IS (except steroids) withdrawal <1month vs 1-3 months | 1.28 | 0.70 – 2.34 | 0.42 |
| IS (except steroids) withdrawal 1-3 months vs > 3 months | 1.68 | 0.85 – 3.34 | 0.43 |
| IS withdrawal 1< month vs > 3 months | 0.76 | 0.39 – 1.49 | 0.43 |
| Steroid withdrawal >3 months vs < 3 months | 0.41 | 0.24 – 0.71 | 0.001 |
| CNI withdrawal < 1 month vs 1-3 months | 1.6 | 0.82 – 3.03 | 0.17 |
| CNI withdrawal < 1 month vs > 3 months | 1.1 | 0.50 – 2.2 | 0.89 |
| CNI withdrawal 1-3 months vs > 3 months | 1.50 | 0.73 – 3.1 | 0.28 |
| MMF/MPA withdrawal < 1 month vs 1-3 months | 1.29 | 0.57 – 2.90 | 0.55 |
| MMF/MPA withdrawal < 1 month vs > 3 months | 1.27 | 0.43 – 3.70 | 0.66 |
| MMF/MPA withdrawal 1-3 months vs > 3 months | 1.01 | 0.30 – 3.10 | 0.98 |

Abbreviation: IS, immunosuppression

Supplementary Table 5

**Description of Infection requiring hospitalization and MACE during the follow-up.**

| **Infection requiring hospitalization** | |
| --- | --- |
| N of patients (%) | 63 (28.9) |
| N of episodes  Bacterial  Viral  Fungal – parasitosis | 115  96  14  5 |
| **Complications cardiovasculaires (MACE)** | |
| N of patients (%) | 18 (8,3) |
| N of episodes  Acute coronary Syndrome  Stroke  Peripheral vascular disease  Sudden death | 21  13  3  4  1 |

Supplementary Table 4

**Univariate analysis of factors associated with the occurrence of infection episodes requing hospitalization during the follow-up after graft failure.**

| Variable | HR | 95% CI (lower- upper) | p-value |
| --- | --- | --- | --- |
| IS (except steroids) withdrawal <1month vs 1-3 months | 1.05 | 0.53 – 2.11 | 0.88 |
| IS (except steroids) withdrawal 1-3 months vs > 3 months | 1.10 | 0.31 – 3.93 | 0.88 |
| IS withdrawal 1< month vs > 3 months | 0.93 | 0.58 – 1.25 | 0.61 |
| Steroid withdrawal >3 months vs < 3 months | 1.24 | 0.70 – 2.20 | 0.48 |
| CNI withdrawal < 1 month vs 1-3 months | 0.76 | 0.42 – 1.35 | 0.35 |
| CNI withdrawal < 1 month vs > 3 months | 0.78 | 0.40 – 1.50 | 0.44 |
| CNI withdrawal 1-3 months vs > 3 months | 0.99 | 0.49 – 1.99 | 0.97 |
| MMF/MPA withdrawal < 1 month vs 1-3 months | 1.00 | 0.62 – 1.64 | 0.97 |
| MMF/MPA withdrawal < 1 month vs > 3 months | 1.00 | 0.60 – 1.70 | 0.90 |
| MMF/MPA withdrawal 1-3 months vs > 3 months | 1.00 | 0.59 – 1.61 | 0.88 |

Supplementary Figure 1

1. Patient survival after graft failure.


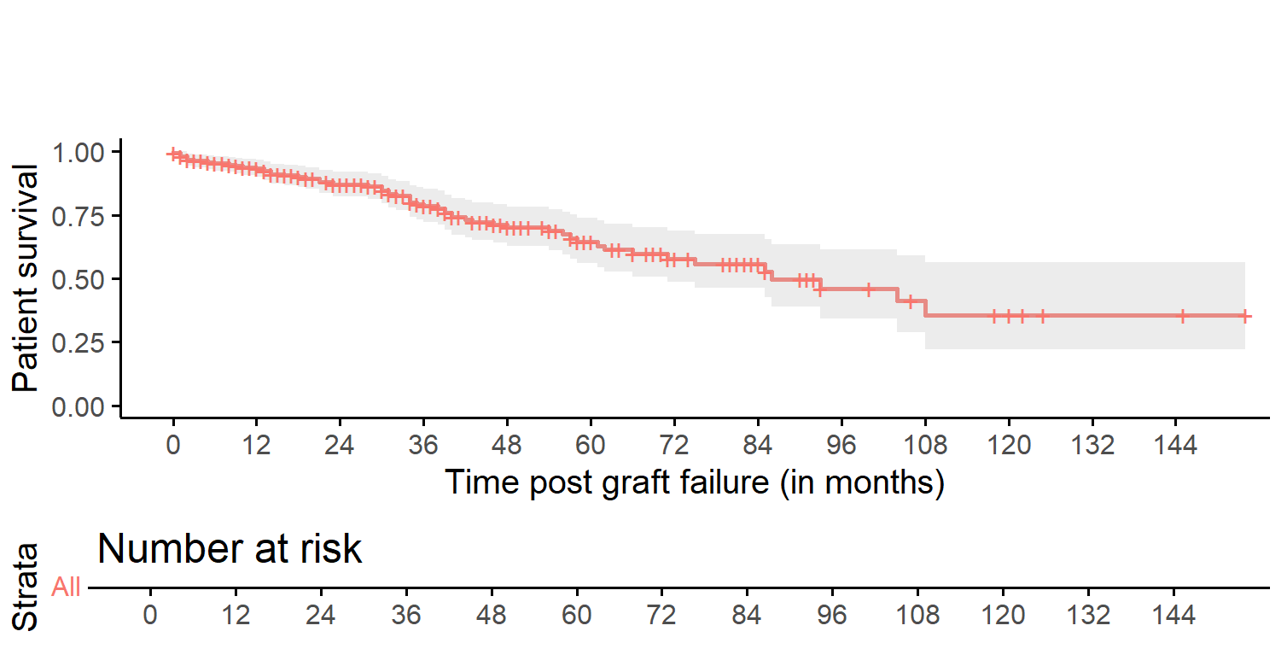


1. Monthly mortality rate after graft failure


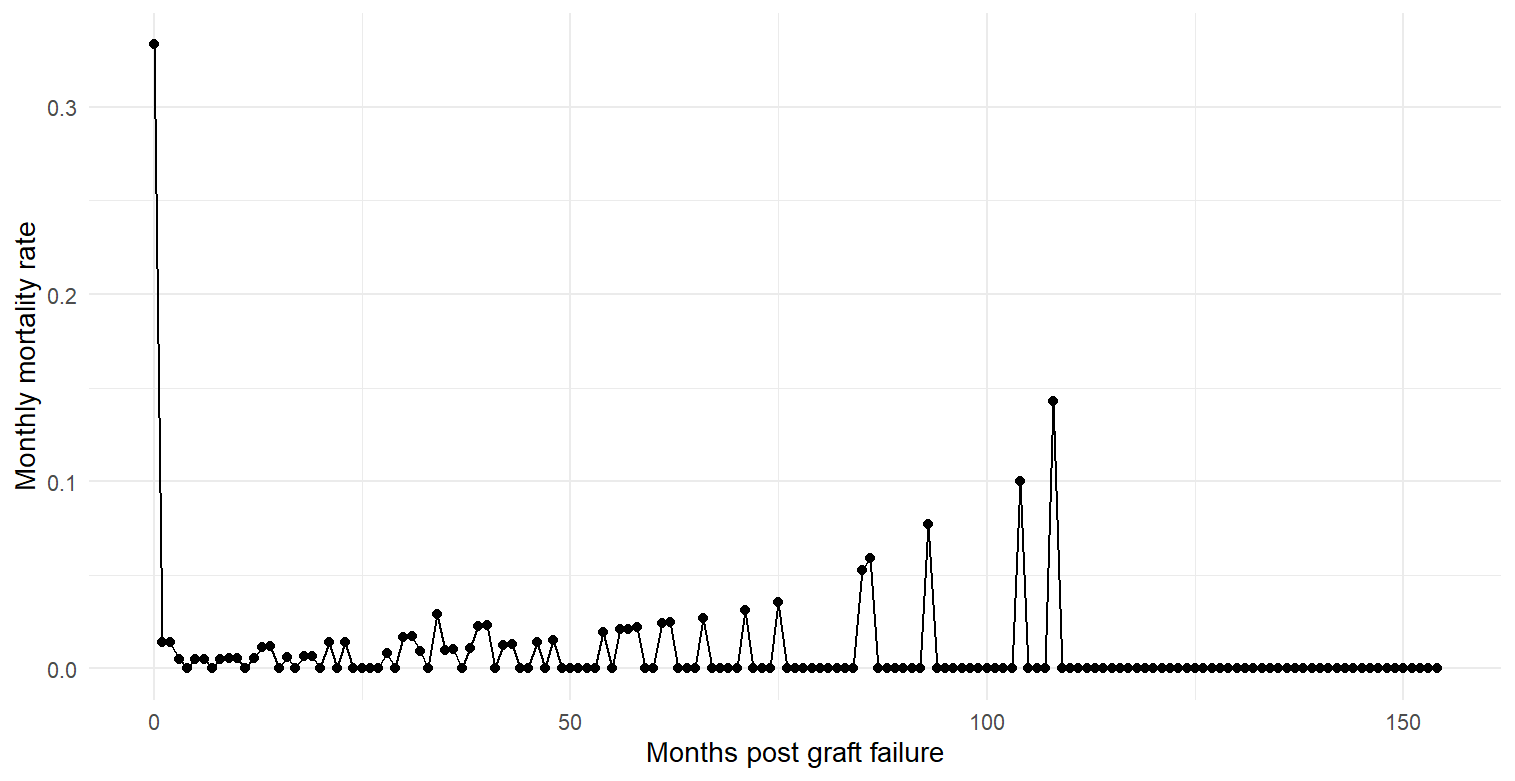


Supplementary Table 6

Causes of death according to the time post graft failure.

| Time post graft failure | Cancer | Infection | Cardiovascular | Unknown |
| --- | --- | --- | --- | --- |
| 0 – 3 months, n(%) | 1 (14.3) | 4 (57.1) | 0 | 2 (28.6) |
| 4 – 12 months n(%) | 0 | 3 (27.3) | 2 (18.2) | 6 (54.5) |
| > 12 months n(%) | 4 (9.7) | 13 (31.7) | 6 (14.6) | 18 (43.9) |

Pearson’s chi-squared test: 0.62
